# Loneliness in Chronic Obstructive Pulmonary Disease: A Multidimensional Determinant of Clinical Outcomes and Disease Management

**DOI:** 10.3390/jcm15103962

**Published:** 2026-05-21

**Authors:** Aminah Mengash, Rayan A. Siraj

**Affiliations:** Department of Respiratory Therapy, College of Applied Medical Sciences, King Faisal University, Al-Ahsa 31982, Saudi Arabia; rsiraj@kfu.edu.sa

**Keywords:** anxiety, chronic obstructive pulmonary disease (COPD), dyspnoea, health-related quality of life (HRQL), loneliness

## Abstract

Chronic obstructive pulmonary disease (COPD) imposes a substantial physical and psychosocial burden, yet the role of loneliness remains under-recognised in clinical practice. Loneliness, defined as a subjective discrepancy between desired and actual social relationships, has emerged as a clinically relevant determinant of patient outcomes. This narrative review synthesises current evidence on the epidemiology, mechanisms, and clinical consequences of loneliness in COPD, and evaluates its implications for disease management. Available evidence indicates that loneliness affects a considerable proportion of individuals with COPD, with prevalence estimates ranging from approximately 18% to over 30%, particularly among patients with greater symptom burden, functional limitation, and oxygen dependence. Dyspnoea and advancing disease severity reduce social participation and increase vulnerability to perceived social disconnection. Loneliness influences COPD outcomes through interconnected behavioural, biological, and healthcare engagement pathways, including systemic inflammation, neuroendocrine stress responses, physical inactivity, impaired self-management, and reduced engagement with healthcare services. These mechanisms contribute to poorer clinical trajectories, as loneliness is consistently associated with reduced health-related quality of life, increased exacerbations, higher healthcare utilisation, greater risk of hospitalisation, and elevated mortality, independent of depression and anxiety. Despite this, loneliness is rarely assessed in routine respiratory care, and targeted interventions remain limited. Emerging strategies, including pulmonary rehabilitation, peer support, and digital health interventions, show promise in reducing loneliness and improving outcomes. Loneliness represents a modifiable and clinically actionable risk factor in COPD, and its integration into routine assessment and management may enhance patient engagement, optimise treatment effectiveness, and reduce healthcare burden. Addressing loneliness represents a critical opportunity to advance more effective and comprehensive COPD care.

## 1. Introduction

Chronic obstructive pulmonary disease (COPD) is a chronic and progressive respiratory condition characterised by persistent airflow limitation and irreversible lung damage [1]. It represents a major global health challenge, affecting nearly 300 million individuals worldwide and ranking among the leading causes of mortality [2]. Beyond its physiological impact, COPD is associated with substantial functional impairment [3]. Symptoms such as dyspnoea, fatigue, and reduced exercise capacity limit daily activities, restrict independence, and often reduce engagement in social life, making COPD a socially and psychosocially limiting disease [4].

Traditionally, COPD research has focused on biological and environmental risk factors, including smoking and air pollution [5,6]. However, there is growing recognition that psychosocial factors also play an important role in shaping disease outcomes. Among these factors, loneliness has emerged as a significant public health concern with potential relevance to chronic respiratory disease [7]. Loneliness has been linked to chronic stress, unhealthy lifestyle behaviours, systemic inflammation, and increased risk of morbidity and mortality in the general population [8]. These pathways closely align with key mechanisms involved in COPD progression, suggesting that loneliness may influence disease outcomes through behavioural, psychological, and biological processes. These overlapping pathways suggest that loneliness may not merely coexist with COPD but may actively contribute to disease expression and progression. In this context, loneliness can be viewed as a clinically relevant factor that interacts with established biological and behavioural determinants of COPD.

Loneliness is defined as a subjective and distressing emotional experience resulting from a perceived lack of meaningful social connection [9,10]. It reflects an individual’s evaluation of the quality and adequacy of their social relationships rather than the number of social contacts alone. This subjective nature distinguishes loneliness as a psychosocial construct that may persist even in the presence of social interaction. As societal and demographic changes continue to reshape social relationships, loneliness is increasingly recognised as a widespread and clinically relevant determinant of health across the life course. In clinical settings, this distinction is particularly important, as loneliness cannot be inferred solely from observable social circumstances and may therefore remain undetected despite regular patient contact. This highlights the need to consider loneliness as a distinct, measurable construct in chronic disease management.

People living with COPD may be particularly vulnerable to loneliness. Persistent respiratory symptoms, physical limitations, and fear of symptom exacerbation can restrict participation in social activities. In addition, disease-related stigma, dependence on healthcare services, and loss of previously held social roles may further contribute to feelings of disconnection [11]. Loneliness in COPD has been associated with poorer quality of life, higher healthcare utilisation, and adverse psychological outcomes, including anxiety and depression [12]. Notably, loneliness is increasingly recognised as a construct that is distinct from, although related to, depression and anxiety, with evidence suggesting that it may exert independent effects on health outcomes through pathways not fully captured by traditional measures of psychological distress. These consequences may create a cycle in which loneliness and disease burden reinforce one another over time.

Despite its clinical relevance, loneliness remains under-recognised in COPD research and practice. Existing studies often focus on broader social factors or psychological comorbidities, with limited attention to loneliness as a distinct construct. Furthermore, the available evidence is fragmented, with many studies relying on cross-sectional designs and offering limited insight into underlying mechanisms or long-term consequences. As a result, loneliness is rarely assessed systematically in routine COPD care, and targeted interventions remain poorly developed. Beyond these gaps, there is a need to better conceptualise how loneliness influences COPD outcomes within a clinically meaningful framework. Loneliness may modify disease burden through multiple interconnected pathways, including behavioural mechanisms (such as reduced physical activity and impaired self-management), biological processes (including stress-related neuroendocrine and inflammatory responses), and healthcare engagement factors (such as reduced participation in rehabilitation and delayed care-seeking). Understanding these pathways is essential for translating existing evidence into clinical practice.

This narrative review aims to synthesise current evidence on loneliness in COPD, focusing on its epidemiology, underlying mechanisms, and clinical consequences. By integrating findings across epidemiological and clinical studies, this review seeks to clarify the role of loneliness in COPD and to identify key gaps that may inform future research and clinical practice.

## 2. Methodology

This narrative review was informed by a structured literature search conducted in PubMed/MEDLINE, Scopus, and Web of Science. Articles published in English were identified using combinations of keywords including COPD, loneliness, social isolation, dyspnoea, pulmonary rehabilitation, and quality of life. The search focused on observational studies, systematic reviews, and meta-analyses addressing the epidemiology, mechanisms, and clinical consequences of loneliness in COPD. Studies were selected based on relevance. Methodological quality and contribution to the conceptual framework of the review.

## 3. Conceptualising Loneliness in Chronic Obstructive Pulmonary Disease

### 3.1. Definition of Loneliness

Loneliness is widely conceptualised as a subjective, distressing emotional experience that arises when an individual perceives a discrepancy between their desired and actual social relationships [11]. This definition highlights that loneliness is not determined by the mere presence or absence of social contacts but by how individuals evaluate the adequacy and meaning of their social connections [13,14]. Deficiencies may therefore be quantitative, such as having few social interactions, or qualitative, such as perceiving existing relationships as emotionally unsatisfying or lacking intimacy. In this sense, loneliness reflects dissatisfaction with social relationships rather than their objective characteristics.

A critical feature of loneliness is its subjective nature. Individuals may experience loneliness even when they are socially active or surrounded by others, while others with limited social contact may not feel lonely. This distinction is central to understanding loneliness as a psychosocial construct that cannot be inferred solely from observable social circumstances. Loneliness is also experienced as emotionally negative and is typically associated with feelings of sadness, emptiness, or disconnection.

Loneliness is distinct from related concepts such as solitude and social isolation. Solitude refers to the voluntary and often positive experience of being alone, which may be restorative rather than distressing. Social isolation, in contrast, describes an objective state characterised by limited social contacts or interactions. While these concepts may overlap, they are not interchangeable. Loneliness captures the internal experience of perceived social inadequacy and may occur independently of social isolation. In this review, loneliness is defined strictly as a subjective experience, and factors related exclusively to social isolation or voluntary solitude are not examined.

Loneliness may be transient, occurring in response to temporary changes such as illness or life transitions, or chronic, when feelings persist over time. Chronic loneliness is of particular concern due to its association with adverse health outcomes and reduced well-being. Recognising these distinctions is essential for understanding loneliness as a meaningful construct in chronic disease contexts, including COPD. In clinical practice, this distinction has important implications, as loneliness may remain undetected in patients who appear socially connected but experience significant subjective disconnection. This highlights the need for clinicians to consider loneliness as a distinct dimension of patient assessment rather than relying solely on observable social circumstances.

Furthermore, conceptualising loneliness as a subjective construct reinforces its potential to influence clinical outcomes independently of related conditions such as depression and anxiety. This distinction is particularly relevant in COPD, where psychological and social factors frequently coexist but may exert differential effects on disease management and prognosis.

### 3.2. Conceptual Frameworks Relevant to Loneliness in COPD

Loneliness is a multifactorial construct shaped by the interaction of individual, health-related, and social factors. Personal characteristics and health status may influence loneliness indirectly through their effects on social roles, psychological stress, and perceived relationship quality. In addition, broader social and cultural contexts shape expectations about relationships and independence, influencing how individuals interpret changes in their social circumstances [15].

In COPD, persistent symptoms such as dyspnoea and fatigue, along with functional limitations, can restrict participation in social activities and disrupt established roles. These changes may reduce engagement in meaningful interactions and alter perceptions of social belonging. Health-related stigma, fear of symptom visibility, and concerns about burdening others may further contribute to social withdrawal [4].

The experience of loneliness in COPD, therefore, reflects not only disease-related limitations but also how individuals perceive and adapt to these changes within their social context. This integrated perspective provides a foundation for understanding the epidemiology, mechanisms, and clinical consequences of loneliness in COPD, as discussed in subsequent sections.

## 4. Prevalence of Loneliness in COPD

Loneliness is increasingly recognised as a major public health concern; however, its prevalence among people living with COPD has been less clearly defined. Although social disconnection has been shown to influence prognosis and treatment response in several chronic diseases, the extent to which loneliness affects individuals with COPD has only recently begun to receive focused attention. As a result, available prevalence estimates remain limited and heterogeneous.

Evidence from observational and synthesis studies suggests that loneliness is common among individuals with COPD. A recent systematic review and meta-analysis, which screened 256 records and included 11 eligible studies comprising 4644 patients with COPD, provides one of the most comprehensive assessments to date [7]. Of the included studies, fewer than half reported quantitative estimates of loneliness or lone living, highlighting inconsistent reporting of outcomes across the literature. Among the five studies included in the meta-analysis, the pooled prevalence of loneliness in COPD was estimated at 32% (95% confidence interval [CI]: 16–48%). A similar prevalence was reported for living alone, at 29% (95% CI: 16–41%). These findings suggest that approximately one in three individuals with COPD experiences loneliness, although substantial variability exists across studies. This heterogeneity likely reflects differences in measurement tools, population characteristics, and study settings. In particular, the use of brief screening measures versus multidimensional scales may capture different aspects of loneliness, limiting direct comparability across studies.

Large-scale population-based evidence further clarifies this burden. Analysis of the nationally representative U.S. Health and Retirement Study (n = 10,384 adults aged ≥ 50 years) is linked to individuals with COPD, who had a significantly higher adjusted prevalence of both social isolation (objective measurement) and loneliness (subjective experience) compared with those without COPD [16]. Loneliness was reported by 18% of individuals with COPD and 22% of those receiving supplemental oxygen, compared with 11% in the non-COPD population. Similarly, social isolation affected 16% of individuals with COPD and 20% of those on oxygen, versus 11% among those without COPD. These data indicate that nearly one in five adults with COPD experience loneliness, with almost double the prevalence observed among those dependent on supplemental oxygen—a subgroup likely characterised by greater functional limitation and visible illness burden. Importantly, loneliness represents a subjective perception of social disconnection, whereas social isolation reflects an objective lack of social contact, and these constructs should not be used interchangeably.

Prospective data further suggest that the relationship between loneliness and COPD may not be unidirectional. In the UK Biobank cohort of nearly 294,000 participants, loneliness was associated with a 31% increased risk of incident COPD [17]. Although this study did not estimate loneliness within established COPD cohorts, it highlights loneliness as a potential psychosocial exposure relevant to disease development, possibly through behavioural pathways such as smoking and inactivity or biological mechanisms including systemic inflammation. These findings raise the possibility that loneliness may act not only as a consequence of COPD but also as a contributing factor in its development, although causal mechanisms remain to be fully established.

Across studies, loneliness in COPD has also been associated with depressive symptoms, difficulty with activities of daily living, multimorbidity, and socioeconomic disadvantage, indicating that both clinical severity and social vulnerability contribute to risk stratification. However, methodological heterogeneity remains a major limitation. The Three-Item UCLA Loneliness Scale is the most used scale, yet other studies rely on single-item measures or proxy indicators, limiting comparability [18]. Most available data are cross-sectional, restricting causal inference and understanding of temporal trajectories. Moreover, residual confounding remains a concern in many studies, as loneliness often coexists with socioeconomic disadvantage, comorbidities, and psychological distress, which may independently influence COPD outcomes.

Taken together, current evidence indicates that loneliness affects a substantial proportion of individuals with COPD, particularly those with greater symptom burden and oxygen dependence. Nevertheless, variability in measurement and study design underscores the need for standardised assessment and longitudinal investigation to more precisely define its epidemiology and clinical significance.

Table 1 summarises key studies investigating loneliness in COPD, including study populations, assessment tools, and reported prevalence estimates or associations. Prevalence values indicate the proportion of participants experiencing loneliness, while hazard ratios (HRs) reflect the risk of incident COPD associated with loneliness.

### 4.1. Dyspnoea as a Clinical Determinant of Loneliness

Dyspnoea, the defining and most distressing symptom of COPD, represents a primary clinical determinant of loneliness [16,19]. While lung function tests quantify airflow limitation, they do not fully capture the lived experience of breathlessness, which is often unpredictable, fluctuating, and emotionally distressing. It is this subjective and activity-limiting dimension of dyspnoea that directly influences social participation. Breathlessness reduces walking capacity, impairs endurance, and limits engagement in routine activities such as shopping, visiting friends, or attending family gatherings. Over time, these restrictions may progressively narrow an individual’s social world. Thus, dyspnoea acts not only as a physiological symptom but also as a constraint on social interaction. In this context, dyspnoea may act as a key interface between physiological impairment and social functioning, linking disease severity to reduced social participation.

Importantly, the clinical relevance of this relationship is supported by commonly used dyspnoea assessment tools. Measures such as the Modified Research Council (MRC) Dyspnoea Scale and the St George’s Respiratory Questionnaire (SGRQ) capture the functional and perceived burden of breathlessness in daily life [20,21,22]. Higher mMRC grades and worse SGRQ scores have been consistently associated with reduced physical activity, impaired health-related quality of life, and decreased social participation in COPD, factors that are closely linked to increased vulnerability to loneliness and social isolation [23,24,25]. These findings suggest that routinely collected dyspnoea measures may provide indirect insights into patients’ social disconnection beyond physical impairment.

Several studies suggest that individuals with COPD may be uniquely vulnerable to social isolation and loneliness because of the high burden of dyspnoea and its functional consequences [19,26,27]. Progressive breathlessness is frequently correlated with reduced mobility and activity avoidance, particularly in community environments that require sustained physical effort. Those with limited mobility—commonly observed in COPD populations due to exertional dyspnoea and lethargy—have been identified as being at increased risk of loneliness. The reduction in physical capacity often results in diminished participation in social roles, including employment, caregiving, and recreational involvement. As these roles contract, opportunities for maintaining interpersonal relationships decline. This gradual disengagement may occur even when social desire remains intact.

Empirical evidence further supports this relationship. In a recent cross-sectional study of elderly patients with COPD, dyspnoea was directly associated with social frailty, a construct reflecting reduced social participation and weakened access to social resources [28]. Structural equation modelling indicated that this association was partly mediated by reduced social support and increased depressive symptoms, suggesting that breathlessness may contribute to social vulnerability through both functional and emotional pathways. Importantly, the direct relationship remained significant after accounting for these mediators. These findings reinforce dyspnoea itself as a central clinical determinant of social disconnection in COPD.

Dyspnoea may also influence loneliness through anticipatory avoidance and altered social confidence. Fear of experiencing breathlessness in public, embarrassment associated with laboured breathing, and concerns about appearing frail or burdensome can discourage participation in social settings. Repeated avoidance behaviours may progressively reduce exposure to meaningful social contact. Over time, diminished interaction may lead to a perceived emotional and social disconnect. Although dyspnoea does not uniformly lead to loneliness, accumulating evidence suggests that greater breathlessness substantially heightens vulnerability to social withdrawal. Accordingly, dyspnoea should be understood not only as a marker of disease severity but as a clinically significant pathway through which loneliness may emerge in COPD.

### 4.2. Disease Severity as a Clinical Determinant of Loneliness

Disease severity represents an important clinical determinant of loneliness in individuals with COPD, although its influence appears to operate primarily through functional limitation and health instability rather than spirometry impairment alone. Population-based evidence from the U.S. Health and Retirement Study demonstrated that individuals with COPD who reported difficulty with one or more activities of daily living were significantly more likely to experience loneliness compared with those without functional difficulty [16]. This suggests that advancing disease burden, particularly when it compromises autonomy, may increase vulnerability to social disconnection.

The same nationally representative analysis showed that individuals with COPD requiring supplemental oxygen—a marker of more advanced disease—had a substantially higher prevalence of loneliness compared with both non-COPD participants and those with COPD not using oxygen. These findings indicate that greater clinical burden is associated with an elevated risk of social isolation and loneliness at a population level.

Additional observational evidence has linked loneliness in COPD to markers of increased healthcare utilisation, including emergency department visits and hospital readmissions [26]. Although these studies do not always stratify directly by GOLD stage or FEV_1_, frequent exacerbations and hospitalisations are widely recognised indicators of more severe and unstable disease. Recurrent acute episodes may disrupt social routines, increase dependence on caregivers, and foster uncertainty about health status, thereby narrowing opportunities for sustained social engagement.

Importantly, however, available studies also demonstrate variability in loneliness across individuals with similar clinical severity. Not all patients with advanced COPD report loneliness, and some individuals with moderate disease experience significant social disconnect [16]. This heterogeneity suggests that disease severity increases risk but does not independently determine loneliness. Rather, its impact appears to be mediated by functional impairment, visible illness burden, comorbid depression, and social context.

Taken together, current evidence indicates that advancing disease burden is associated with higher loneliness risk in COPD, particularly when it compromises functional independence. However, physiological severity alone does not fully account for social outcomes, underscoring the need for multidimensional assessment.

## 5. Pathways Linking Loneliness to Clinical Outcomes

**Figure 1:**
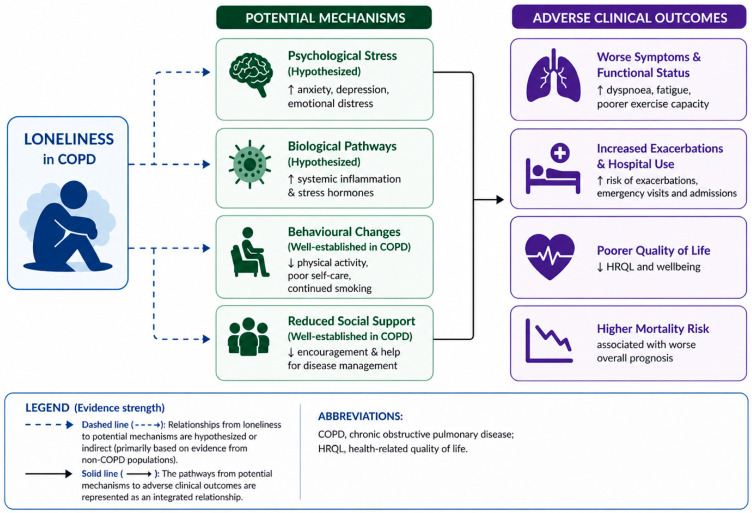
**Conceptual model illustrating pathways linking loneliness to adverse clinical outcomes in COPD.** Loneliness may contribute to adverse outcomes through interconnected psychological, biological, behavioural, and social pathways, potentially leading to worse symptoms and functional status, increased healthcare utilisation, poorer quality of life, and higher mortality risk.

Loneliness may act as a central psychosocial stressor that influences psychological distress, biological processes such as systemic inflammation and stress hormone activation, and behavioural changes including reduced physical activity and poor self-management. It is also associated with reduced social support, which further limits disease management. Together, these mechanisms lead to worse symptoms and functional status, increased exacerbations and healthcare utilisation, poorer quality of life, and higher mortality risk. Figure 1 illustrates a conceptual model of how loneliness in COPD contributes to adverse clinical outcomes through multiple interconnected pathways. Importantly, these pathways should not be viewed as independent processes but as interconnected mechanisms through which loneliness may interact with existing disease-related factors in COPD. In this context, loneliness may amplify physiological stress responses, influence health behaviours, and alter patterns of healthcare engagement, thereby contributing to adverse clinical outcomes.

### 5.1. Biological Mechanisms

#### 5.1.1. Inflammatory Activation

Chronic exposure to cigarette smoke, environmental pollutants, or other inhaled foreign particles initiates the inflammatory cascade that underlies COPD. These stimuli activate airway epithelial cells and resident immune cells, triggering the release of cytokines and chemokines that recruit circulating leukocytes and sustain airway inflammation. As the disease progresses, distinct inflammatory phenotypes emerge, contributing to clinical heterogeneity [29]. While these inflammatory processes are primarily driven by inhalational exposures, emerging evidence suggests that psychosocial stressors such as loneliness may modulate systemic inflammatory activity, potentially amplifying disease-related inflammation.

In neutrophilic COPD, epithelial injury activates macrophages and promotes the release of proinflammatory mediators such as CXCL8 (IL-8), IL-6, G-CSF, and leukotriene B4 (LTB4), leading to neutrophil recruitment. Neutrophils play a central role in disease pathogenesis by releasing proteolytic enzymes (e.g., neutrophil elastase, matrix metalloproteinase-9, MMP-9) and reactive oxygen species, which degrade extracellular matrix, disrupt epithelial integrity, and impair mucociliary clearance. This protease–antiprotease imbalance contributes to airway remodelling and emphysema [30]. Clinically, this phenotype is associated with more severe airflow limitation, increased sputum production, hypoxemia, and reduced responsiveness to corticosteroids. Systemic inflammation is reflected by elevated biomarkers such as C-reactive protein (CRP) and neutrophil to lymphocyte ratio, which are linked to increased exacerbation risk and mortality [31].

In contrast, eosinophilic COPD arises from a T-helper 2 (Th2) mediated response involving cytokines such as IL-5 and IL-13. This phenotype is associated with milder airflow limitation and better response to corticosteroid therapy. Elevated levels of eosinophil counts in blood or sputum serve as clinically useful biomarkers for exacerbation risk and treatment responsiveness [32,33].

Overall, persistent airway and systemic inflammation contribute to disease progression and broader systemic effects in COPD. In the context of loneliness, chronic inflammatory activation may represent a potential biological pathway linking psychosocial stress to adverse clinical outcomes; however, this relationship is largely inferred from general psychoneuroimmunology research rather than COPD-specific evidence, and should therefore be considered hypothesised rather than definitively established in COPD populations. Emerging prospective evidence further supports a potential temporal relationship between loneliness and COPD. Data from the UK Biobank cohort indicate that loneliness is associated with an increased risk of incident COPD (HR: 1.31; 95% CI: 1.20–1.43), suggesting that loneliness may act as a behavioural or biological “priming” factor through pathways such as smoking, physical inactivity, and systemic inflammation. Nevertheless, the underlying mechanisms in COPD populations remain incompletely understood and require further investigation.

#### 5.1.2. Neuroendocrine Stress System

COPD is associated with dysregulation of neuroendocrine stress symptoms, particularly the hypothalamic–pituitary–adrenal (HPA) axis and the sympathetic-adrenal-medullary (SAM) system. Chronic physiological stressors, including systemic inflammation, recurrent exacerbation, hypoxemia, and hypercapnia, lead to sustained activation of these pathways. Loneliness has been consistently associated with chronic activation of stress-response systems in non-COPD populations, suggesting that it may act as a persistent psychosocial stressor capable of exacerbating these neuroendocrine processes. This results in altered secretion of stress hormones such as corticotropin-releasing hormone (CRH), adrenocorticotropic hormone (ACTH), cortisol, and catecholamines [34].

Prolonged activation of the HPA axis may contribute to dysregulation characterised by either elevated cortisol levels or impaired cortisol responsiveness. In parallel, inflammatory mediators such as IL-6, TNF-α, and CRP interfere with glucocorticoid receptor function, contributing to glucocorticoid resistance and further amplifying systemic inflammation. Gas exchange abnormalities further exacerbate this process. Chronic hypoxemia increases oxidative stress and neuronal injury, while hypercapnia disrupts central nervous system homeostasis and neurotransmitter regulation. Together, these factors create a state of persistent physiological stress and neuroendocrine imbalance [35]. This dysregulation represents a key mechanism linking COPD to cognitive impairment and psychological vulnerability. Importantly, loneliness may intensify these stress responses, reinforcing a cycle of inflammation, neuroendocrine dysfunction, and adverse clinical outcomes. This highlights a potential bidirectional interaction, where COPD-related physiological stress and loneliness may reinforce each other, contributing to cumulative disease burden.

### 5.2. Behavioural Mechanisms

#### 5.2.1. Physical Inactivity and Deconditioning

Physical inactivity is a common yet often under-recognised behavioural issue in COPD and is independently associated with adverse health outcomes. While interventions such as pulmonary rehabilitation and pedometer-based self-monitoring can improve activity levels in the short term, sustaining long-term behavioural change remains challenging. Effective strategies require patient-centred approaches that consider individual symptoms, functional limitations, confidence, and self-management capacity [36]. In the context of loneliness, these challenges may be further amplified, as reduced social support, diminished motivation, and lack of external encouragement can negatively influence engagement in physical activity.

Qualitative evidence indicates that patients with COPD face multiple barriers to physical activity, including fear of breathlessness, low motivation, environmental constraints, and limited social support. Loneliness may intensify these barriers by increasing the perceived effort associated with physical activity, reducing accountability, and limiting opportunities for socially reinforced behaviours, such as group-based exercise or participation in rehabilitation. These barriers contribute to inactivity independently of disease severity and highlight the need for comprehensive behavioural interventions [37].

Inactivity accelerates physical deconditioning, reduces exercise tolerance, and reinforces sedentary behaviour. Importantly, physical activity has been identified as a strong predictor of survival; in a prospective cohort of patients with stable COPD, higher activity levels were associated with significantly lower risk of all-cause mortality (HR 0.46; 95% CI, 0.33–0.64; *p* < 0.001) [38]. These findings suggest that behavioural pathways, particularly physical inactivity, may represent a key mechanism through which loneliness contributes to adverse outcomes in COPD.

Reduced physical activity also contributes to skeletal muscle dysfunction, including loss of muscle mass, reduced strength, and impaired endurance. Approximately 40% of patients experience exercise limitation primarily due to peripheral muscle abnormalities rather than pulmonary impairment. These changes—characterised by muscle atrophy, fibre-type shifts, and reduced oxidative capacity—are driven by factors such as disuse, hypoxemia, malnutrition, oxidative stress, and systemic inflammation, ultimately worsening functional capacity and quality of life [39]. Taken together, these findings indicate that physical inactivity in COPD is not solely a consequence of physiological limitation but may also be influenced by psychosocial factors such as loneliness. This reinforces the role of inactivity as a mediating pathway linking loneliness to functional decline, reduced quality of life, and increased mortality risk.

#### 5.2.2. Impaired Self-Management Behaviors

Effective self-management in COPD involves symptom monitoring, medication adherence, and lifestyle modification. Cognitive impairment is a key barrier, with evidence showing that even mild deficits can negatively affect daily self-care. In the context of loneliness, these challenges may be further compounded, as reduced social support and limited interpersonal reinforcement can hinder the development and maintenance of effective self-management behaviours. A systematic review reported that cognitive decline in COPD is associated with poorer inhaler technique, reduced symptom recall, and increased dependence in daily activities. Executive dysfunction and dyspraxia emerging as key predictors of inhaler incompetence [40].

Health literacy is another critical determinant of self-management. Patients with limited health literacy demonstrate significantly lower medication adherence, even when caregiver support is available (OR 0.44; 95% CI: 0.24–0.81) [41]. Similarly, impairments in fluid cognitive abilities—such as working memory and executive function—are linked to poor adherence and incorrect inhaler use, whereas crystallised knowledge appears less influential [42]. Loneliness may exacerbate these challenges by limiting access to informational support, reducing opportunities for clarification of health-related information, and weakening engagement with healthcare providers.

Behavioural models further highlight the complexity of self-management. According to the Information–Motivation–Behavioural Skills (IMB) model, patients may struggle due to insufficient knowledge, low motivation, or inaccurate health beliefs. Even motivated individuals may rely on incorrect information, undermining effective disease management [43]. In this framework, loneliness may act as a critical modifier by reducing motivation, increasing psychological burden, and limiting the social context necessary for reinforcing positive health behaviours.

Taken together, these findings suggest that impaired self-management in COPD is not solely driven by cognitive or informational deficits but may also be significantly influenced by psychosocial factors such as loneliness. This highlights self-management as a key behavioural pathway through which loneliness may contribute to adverse clinical outcomes.

#### 5.2.3. Reduced Engagement with Healthcare and Rehabilitation

Pulmonary rehabilitation (PR) is a cornerstone of COPD management; however, participation and adherence remain suboptimal. Barriers include symptom burden, low motivation, limited awareness, and psychosocial factors. Loneliness may represent a key psychosocial barrier in this context, as individuals experiencing social disconnection may have reduced motivation, limited encouragement, and fewer opportunities for engagement with structured rehabilitation programs. Certain subgroups, such as underweight patients, face additional challenges due to lower baseline exercise capacity, reduced muscle strength, and poorer quality of life, despite demonstrating benefit from PR programs [44].

Adherence to rehabilitation is influenced by multiple factors, including patient capability (self-management skills), opportunity (access to resources and professional support), and motivation (psychological factors). Evidence from a systematic review highlights the importance of individualised and supportive approaches to improve engagement [45]. Within this framework, loneliness may adversely affect all three components by limiting social support (opportunity), reducing confidence and self-management capacity (capability), and diminishing motivation to engage in sustained rehabilitation behaviours.

Non-attendance and poor adherence are common. A large retrospective study reported that nearly one-third of patients did not attend PR, and a similar proportion were non-adherent. Key predictors included smoking status, living alone, and greater severity, indicating that both social and clinical factors play a role in limiting engagement [46]. The association between living alone and reduced rehabilitation engagement further supports the role of social disconnection as a determinant of healthcare utilisation in COPD. In this context, loneliness may contribute to delayed care-seeking, reduced adherence to recommended interventions, and lower participation in structured programs such as pulmonary rehabilitation.

## 6. Clinical Consequences of Loneliness in COPD

Loneliness is increasingly recognised as an independent determinant of adverse outcomes in COPD, affecting both clinical and psychosocial domains. These outcomes reflect the cumulative impact of behavioural, biological, and healthcare engagement pathways through which loneliness may influence disease progression and patient experience.

### 6.1. Health-Related Quality of Life (HRQL)

Loneliness is consistently associated with poorer health-related quality of life (HRQL) in COPD. HRQL declines with increasing disease severity, with patients in higher Global Initiative for Chronic Obstructive Lung Disease (GOLD) stages demonstrating worse scores across all domains of the St George’s Respiratory Questionnaire (SGRQ), reflecting greater functional limitation and reduced well-being [47]. However, loneliness may further modify this relationship by influencing patients’ perception of their health status and social functioning, beyond what is explained by disease severity alone.

Beyond disease severity, HRQL is influenced by multiple factors, including age, socioeconomic status, treatment burden, comorbidities, and medication adherence [48]. Psychosocial factors, particularly loneliness, further exacerbate impairment in quality of life, especially in emotional and social domains. This suggests that loneliness may disproportionately affect subjective dimensions of health, including emotional well-being and perceived social participation. Importantly, interventional studies indicate that structural social and psychological support can significantly improve HRQL, as well as dyspnoea, exercise capacity, mental health, and lung function, particularly in patients with mild to moderate disease [49]. These findings indicate that addressing loneliness may offer additional benefits beyond traditional clinical management, particularly by improving patient-reported outcomes.

### 6.2. Exacerbations and Healthcare Utilisation

Social isolation (objective condition) and loneliness (a subjective experience) are both common in COPD; however, they have been associated with poorer disease management and reduced treatment adherence. These factors contribute to an increased risk of exacerbation and acute clinical deterioration [16]. These associations are likely mediated through behavioural pathways, including poor adherence, delayed symptom recognition, and reduced engagement with preventive care.

Loneliness has also been independently associated with worse health perception, greater breathlessness, reduced exercise capacity, and higher rates of emergency department visits and healthcare utilisation in patients with moderate-to-severe COPD (OR = 1.57, 95% CI: 1.005–2.466) [26,50]. These findings highlight that addressing loneliness not only reflects psychosocial distress but also contributes directly to increasing healthcare demand. This reinforces the importance of considering loneliness within clinical risk stratification, particularly in patients with frequent exacerbations or high healthcare utilisation.

### 6.3. Hospitalisation and Readmission

Hospitalisation rates are high in COPD, particularly among patients with frequent exacerbations and multiple comorbidities. Loneliness may further increase this burden by impairing self-management, reducing adherence to treatment, and delaying care-seeking behaviours.

Evidence from large cohort studies shows that patients with COPD experience high rates of emergency department visits and hospital admissions, with repeated admission strongly associated with disease complexity (OR up to 4.3) [51]. Within this context, loneliness acts as an additional risk factor, compounding clinical vulnerability with psychosocial disadvantage. This combination increases the likelihood of hospitalisation and potentially readmission, highlighting the need for integrated care approaches addressing social factors. In this context, loneliness may act as a compounding factor that amplifies existing clinical vulnerability, increasing the likelihood of hospital-based care.

### 6.4. Mortality and Prognostic Implications

Loneliness has emerged as an independent predictor of mortality and adverse prognosis in COPD. Its impact extends beyond traditional clinical risk factors and operations through behavioural, psychological, and biological pathways.

Patients experiencing loneliness are more likely to demonstrate poor treatment adherence, reduced participation in PR, and delayed healthcare utilisation, all of which increase the risk of exacerbations and hospitalisation [52]. These behavioural factors may partially explain the observed association between loneliness and mortality risk. In addition, chronic activation of stress pathways may lead to sustained low-grade systemic inflammation reflected by elevated biomarkers such as IL-6 and CRP, contributing to disease progression [53]. However, it should be noted that most available evidence is observational, and further longitudinal studies are needed to establish causal pathways. Collectively, these findings highlight loneliness as a clinically relevant determinant of prognosis in COPD, underscoring the need for its routine assessment and integration into comprehensive disease management strategies.

### 6.5. Independent Effects of Loneliness Beyond Depression and Anxiety

Loneliness is a distinct construct that exerts independent effects on health outcomes in COPD, separate from depression and anxiety. Although these conditions frequently coexist, loneliness remains significantly associated with poorer HRQL, increased symptom burden, and greater healthcare utilisation even after adjustment for mood disorders. This suggests that loneliness captures a unique dimension of patient experience that is not fully explained by traditional psychological constructs.

These independent effects are likely mediated through mechanisms not fully captured by depression and anxiety, including impaired self-management, reduced social support, and physiological pathways such as increased stress reactivity and low-grade inflammation [54,55,56]. This distinction has important clinical implications, as it supports the need to assess and address loneliness as a separate component of COPD management rather than as a secondary feature of psychological comorbidity.

## 7. Loneliness, Self-Management, and Rehabilitation

### 7.1. Medication Adherence and Inhaler Technique

Although loneliness has not been extensively studied as a primary determinant of inhaler adherence in COPD, poor self-management, incorrect inhaler technique, and medication non-adherence are well-documented barriers to effective disease control [57]. Emerging evidence suggests that loneliness may indirectly influence these behaviours.

Longitudinal data in chronic disease populations indicate that loneliness mediates the relationship between social isolation and medication adherence, alongside social support, highlighting the importance of social connectedness in maintaining treatment routines [58]. In COPD, loneliness may reduce motivation, limit access to accurate health information and support, thereby reinforcing existing misconceptions about medications and negatively affecting adherence and inhaler technique [59].

In this context, loneliness may act as a behavioural barrier by reducing accountability, limiting reinforcement of correct inhaler use, and diminishing adherence to prescribed treatment regimens. Taken together, these findings suggest that medication adherence and inhaler technique represent important pathways through which loneliness may contribute to suboptimal disease control in COPD.

### 7.2. Pulmonary Rehabilitation Uptake, Participation, and Completion

PR is the cornerstone of COPD management; however, referral, uptake and completion remain suboptimal, particularly in low- and middle-income settings [60]. Barriers to participation are multifactorial and include transportation difficulties, personal prejudice of health, lack of motivation, and exacerbations [61]. In this context, loneliness may act through behavioural and motivational pathways by reducing accountability, limiting social reinforcement, and increasing perceived effort associated with participation in rehabilitation programs. Given that PR is typically delivered in group settings, individuals experiencing loneliness may be less inclined to engage in or sustain participation in socially interactive environments, further contributing to reduced uptake and completion.

These findings suggest that loneliness may represent a clinically relevant barrier to pulmonary rehabilitation, linking psychosocial factors with reduced treatment engagement and suboptimal clinical outcomes.

### 7.3. Self-Efficacy and Confidence in Disease Management

Self-efficacy—the confidence in a patient’s ability to manage the disease—is a central component of effective COPD self-management. Loneliness can undermine self-efficacy by reducing social support, motivation, and confidence in managing medications, inhaler technique, and lifestyle changes [62].

Emerging evidence suggests that emotional intelligence is important in COPD management. Higher emotional intelligence is associated with better self-management behaviours and improved quality of life, independent of disease severity [63]. Loneliness may negatively influence these factors by increasing psychological burden, reducing emotional resilience, and limiting opportunities for social reinforcement of positive health behaviours. In addition, structural interventions such as patient education and motivational interviewing, delivered through a multidisciplinary approach, have been shown to improve disease knowledge, treatment adherence, and survival outcomes [64].

Taken together, these findings suggest that loneliness may impair self-efficacy, representing a key pathway through which psychosocial factors influence self-management behaviours and clinical outcomes in COPD.

## 8. Assessment and Interventions in Clinical Practice

### 8.1. Tools Available for Assessing Loneliness in COPD Care

Assessment of loneliness in COPD has gained increasing attention due to its impact on clinical outcomes. Several validated tools are available for use in both research and clinical settings. The UCLA Loneliness Scale (Version 3) is one of the most widely used instruments for assessing subjective loneliness and perceived social isolation, with well-established reliability and validity [65]. Its brief 3-item version is particularly suitable for clinical use and large-scale studies, capturing core dimensions of loneliness—lack of companionship, feeling left out, and feeling isolated from others—with good psychometric performance [66]. Importantly, these tools assess subjective perceptions of social disconnection, which may differ from the objective of socialization [7]. This distinction is particularly important in COPD care, where patients may appear socially connected yet experience significant subjective loneliness that remains clinically unrecognised.

Other tools include the De Jong Gierveld Loneliness Scale, which provides a multidimensional assessment by distinguishing between emotional loneliness (absence a of close relationship) and social loneliness (lack of a broader social network), and has been validated across diverse populations [66,67]. In COPD populations, loneliness is often assessed alongside measures of psychological well-being, such as depression and anxiety (e.g., PHQ-9, GAD-7), reflecting the multidimensional nature of social well-being [68,69]. Brief tools such as the 3-item UCLA scale have been effectively used in clinical and emergency care settings and have been associated with outcomes such as increased healthcare utilisation [26,68].

Importantly, higher loneliness scores—particularly when assessed using brief tools such as the 3-item UCLA scale—have been associated with adverse clinical outcomes, highlighting the clinical relevance of incorporating loneliness screening into routine COPD assessment. Despite their availability, these tools are not routinely integrated into COPD care frameworks, underscoring a gap between evidence and clinical practice.

### 8.2. Limitations and Feasibility of Screening in Routine Respiratory Practice

Despite the availability of validated tools, routine screening for loneliness in respiratory care remains limited. Several barriers affect feasibility in clinical practice. Time constraints in busy outpatient settings often restrict the inclusion of psychosocial assessments. In addition, healthcare professionals may lack training or confidence in addressing loneliness, and the absence of clear referral pathways following identification further limits implementation [70,71].

Loneliness is inherently subjective and dynamic, and may fluctuate over time, making it difficult to capture through single assessments [72]. Most tools rely on self-report, which may lead to underreporting due to stigma or social desirability bias. Furthermore, social health indicators are not routinely integrated into COPD management frameworks, reducing prioritisation in clinical care. As a result, loneliness may remain systematically under-recognised despite its demonstrated association with adverse clinical outcomes.

Nevertheless, brief and validated tools, such as the 3-item UCLA Loneliness Scale, offer a feasible option for rapid screening and may be integrated into routine practice when combined with appropriate clinical pathways [73]. Integrating loneliness screening into routine care will require not only practical tools but also clear clinical pathways to guide intervention following identification.

### 8.3. Interventions Addressing Loneliness

#### 8.3.1. Pulmonary Rehabilitation as a Social and Behavioural Intervention

Pulmonary rehabilitation PR is a key component of COPD management and provides benefits beyond physical improvement. PR has been shown to enhance health-related quality of life, emotional well-being, anxiety, and depression. It is a group-based format, and inclusion of psychological support fosters social interaction, a sense of belonging, and mutual encouragement.

Evidence suggests that loneliness influences PR outcomes. Higher baseline loneliness is linked to poorer physical and psychological status; however, patients who experience a reduction in loneliness during PR demonstrate greater improvement in exercise capacity and quality of life [74]. At the same time, loneliness may act as a barrier to PR uptake and adherence by reducing motivation and increasing psychological distress more broadly. Barriers to PR participation are multifactorial and include behavioural, social, and system-level factors such as limited access and low referral rates. Importantly, while PR and related interventions may support social engagement, direct evidence demonstrating specific reductions in loneliness among COPD populations remains limited and should be interpreted cautiously.

#### 8.3.2. Peer Support and Group-Based Approaches

Peer support interventions provide opportunities for patients to connect with others who share similar experiences, promoting social engagement and emotional support. In COPD, these approaches are often integrated into self-management programmes.

Evidence from a randomised study suggested that peer support can improve patient engagement and reduce healthcare utilisation [75]. Broader evidence across chronic diseases found that such interventions enhance self-management, emotional well-being, and social connectedness, while addressing loneliness and isolation [76].

#### 8.3.3. Digital and Telehealth-Based Strategies

Digital health interventions are increasingly used to address barriers in COPD, particularly for patients with mobility limitations and geographic isolation. Approaches such as telerehabilitation, virtual support groups, and mobile health (mHealth) applications allow remote access to care while maintaining elements of social interaction. These approaches may be particularly relevant for individuals experiencing loneliness, as they provide opportunities for connection while overcoming physical and logistical barriers.

Evidence suggests that telerehabilitation can be as effective as centre-based programmes, with additional benefits in functional capacity, self-efficacy, mental health, and healthcare utilisation. However, the current evidence base remains limited by small sample sizes and heterogeneity of studies.

**Figure 2:**
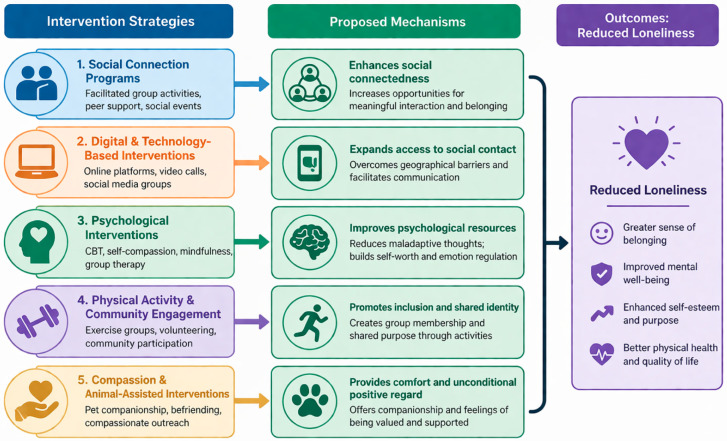
**Intervention strategies targeting loneliness and their proposed mechanisms.** The figure illustrates key interventions that may reduce loneliness in COPD, including pulmonary rehabilitation, peer support, and digital or telehealth-based approaches. These strategies may act through several overlapping mechanisms, such as enhancing social connectedness, improving self-efficacy and motivation, increasing engagement with self-management and rehabilitation, and reducing barriers related to mobility limitations or restricted access to care. Through these pathways, interventions targeting loneliness may contribute to improved health-related quality of life, better treatment engagement, and more favourable clinical outcomes.

Challenges to implementation include low digital literacy, limited access to technology, and variable patient engagement. Qualitative evidence indicates that the successful use of digital interventions depends on factors such as health literacy, user experiences, motivation, and social support [77]. Furthermore, long-term effectiveness, particularly in addressing loneliness, requires further investigation (Figure 2).

## 9. Clinical Implications, Research Gaps, and Conclusion

### 9.1. Loneliness as a Modifiable and Clinically Relevant Risk Factor

Loneliness is increasingly recognised as a prevalent and clinically significant risk factor in COPD, affecting up to one in five patients and occurring more frequently than in the general population [16]. It is associated with poorer functional exercise capacity, particularly among patients undergoing pulmonary rehabilitation [74]. In addition, loneliness has been independently linked to higher rates of emergency department visits and poorer health perception, even after adjustment for disease severity and other clinical variables [26]. These findings position loneliness as a clinically relevant determinant that extends beyond psychosocial burden, influencing functional status and healthcare utilisation.

Importantly, loneliness is modifiable. Evidence indicates that reductions in loneliness are associated with improvements in exercise capacity and quality of life, particularly within pulmonary rehabilitation programmes. Longitudinal data further suggested that persistent loneliness may increase the risk of developing COPD and contribute to disease progression [78]. Systematic reviews also demonstrate its negative impact on treatment response and overall prognosis [7]. Together, these findings support the integration of loneliness assessment and targeted psychosocial interventions into routine COPD care. From a clinical perspective, this highlights an opportunity to intervene on a potentially reversible factor that is not currently addressed within standard COPD management frameworks.

#### 9.1.1. Conceptual Role of Loneliness in COPD

Loneliness in COPD should be interpreted within a structured conceptual hierarchy. First, loneliness may act as a risk factor, contributing to behavioural and biological changes such as physical inactivity, smoking, and systemic inflammation that may influence disease development and progression. Second, loneliness may serve as a prognostic marker, as it has been consistently associated with poorer clinical outcomes, including increased exacerbations, healthcare utilisation, and reduced health-related quality of life. Third, loneliness may function as a mechanistic driver, influencing disease outcomes through interconnected psychological, behavioural, and biological pathways. However, the strength of evidence differs across these roles, with most available data derived from observational studies. Therefore, these roles should be interpreted with caution, and causal inferences remain limited.

#### 9.1.2. Reverse Causality and Bidirectional Relationships

The relationship between loneliness and clinical outcomes in COPD is likely bidirectional rather than unidirectional. While loneliness has been associated with adverse outcomes, it is also plausible that worsening disease severity contributes to increased loneliness. Greater dyspnoea burden, higher exacerbation frequency, and worsening clinical status have been shown to correlate with increased loneliness in COPD populations [79]. In addition, persistent breathlessness and functional limitation may restrict social participation and contribute to social withdrawal, thereby increasing perceived loneliness [80]. This is further supported by longitudinal evidence, which suggests that early disease manifestations, such as reduced exercise tolerance and dyspnoea, may contribute to loneliness, highlighting the potential for reverse or bidirectional causality [78]. Moreover, loneliness itself has been associated with increased exacerbation risk and poor clinical outcomes, reinforcing the complex interplay between psychosocial and clinical factors in COPD [50].

### 9.2. Integration of Loneliness into COPD Care Pathways

Incorporating loneliness into COPD care requires moving beyond screening toward risk stratification and personalised intervention strategies. Emerging evidence indicates that loneliness is not only a consequence of COPD but also a predictor of disease development. Large cohort data show that loneliness is associated with an increased risk of incident COPD, partly mediated by modifiable behaviour such as smoking, physical inactivity, and poor diet [17]. This reinforces the need to consider loneliness not only as a downstream consequence of disease but also as an upstream determinant influencing disease trajectory.

Integrating loneliness into multidimensional care models can improve outcomes by addressing social, behavioural, and clinical factors simultaneously. Limited social support has been associated with reduced mobility, poorer self-management, and diminished quality of life [81]. Interventional studies further demonstrate that structured psychosocial support can improve exercise capacity, mental health, and overall well-being [49]. However, these interventions are rarely structured around loneliness as a primary target, limiting their potential effectiveness in addressing social disconnection.

Embedding loneliness within dynamic care pathways—where it is routinely monitored alongside clinical indicators—may facilitate targeted interventions such as pulmonary rehabilitation, social prescribing, and digital support. This approach reflects a shift toward a proactive biopsychosocial model of care and may reduce healthcare utilisation while improving patient outcomes [50]. Operationalising this approach will require integration into routine assessment, clear referral pathways, and multidisciplinary collaboration.

### 9.3. Implications for Clinical Guidelines and Service Design

The integration of loneliness into COPD clinical guidelines requires its recognition as a core clinical indicator rather than an optional psychosocial factor. Current evidence shows that loneliness affects not only quality of life but also clinical endpoints such as hospitalisations, exacerbations, and response to pulmonary rehabilitation, indicating that it should be treated similarly to other risk factors like smoking or physical inactivity. Failure to address loneliness may therefore limit the effectiveness of existing clinical interventions.

From a service design perspective, care models should adopt integrated biopsychosocial approaches that embed loneliness interventions into existing COPD services. One promising strategy is social prescribing, where patients are referred to community-based activities, peer support, or behavioural programmes. Evidence suggested that such approaches can reduce loneliness and improve well-being, although standardisation remains a challenge [82]. Embedding such approaches within COPD services may enhance both patient engagement and long-term outcomes.

### 9.4. Key Research Gaps and Future Directions

Despite growing recognition, several important research gaps remain. First, prevalence estimates vary due to inconsistent measurement tools, highlighting the need for standardised assessment methods. Second, the mechanisms linking loneliness to COPD outcomes—such as inflammation, physical activity, and impaired self-management—require further investigation through longitudinal and mechanistic studies. In particular, the relative contribution of behavioural, biological, and healthcare engagement pathways remains poorly defined.

Finally, evidence for intervention remains limited. While pulmonary rehabilitation, peer support, and digital programs show promise, few studies specifically target loneliness as a primary outcome, and long-term effectiveness and scalability are unclear. Future research should prioritise a high-quality trial, standardised measurement, and integrated intervention models. In addition, future studies should evaluate loneliness as a primary outcome rather than a secondary or exploratory measure.

### 9.5. Limitation

This review has several limitations. First, substantial heterogeneity exists in the measurement of loneliness across studies, with variations in tools such as the UCLA Loneliness Scale, brief screening instruments, and single-item measures, limiting comparability of findings. Second, most of the available evidence is derived from observational studies, which are inherently subject to residual confounding and cannot establish definitive causal relationships. Important factors such as disease severity, comorbidities, and socioeconomic status may influence both loneliness and clinical outcomes. Third, the predominance of cross-sectional data limits the ability to determine temporal directionality, and bidirectional or reverse causality remains likely. Finally, although loneliness appears to exert effects independent of depression and anxiety, overlap between these constructs may not be fully accounted for across all studies. These limitations should be considered when interpreting the current evidence base.

## 10. Conclusions

Loneliness is a common and clinically significant factor in COPD that adversely affects physical health, psychological well-being, and overall quality of life. Beyond its psychosocial impact, accumulating evidence indicates that loneliness influences key clinical outcomes through behavioural, biological, and healthcare engagement pathways, contributing to poorer disease control and increased healthcare utilisation.

As a modifiable risk factor, loneliness represents an important and under-recognised target for intervention. Strategies such as pulmonary rehabilitation, peer support, and digital health interventions offer promising approaches to reducing loneliness while simultaneously improving self-management behaviours and clinical outcomes. However, these interventions are not consistently designed or implemented with loneliness as a primary focus, highlighting an important gap in current COPD care.

Integrating loneliness assessment and management into routine clinical practice has the potential to enhance patient engagement, optimise treatment effectiveness, and support a more comprehensive, patient-centred approach to care. This requires moving beyond recognition toward structured implementation, including routine screening, clear referral pathways, and multidisciplinary interventions targeting social as well as clinical needs.

Recognising and addressing loneliness in COPD is therefore essential for improving patient outcomes and advancing the quality of care. It represents a critical step toward a more holistic model of disease management that integrates clinical, behavioural, and social dimensions of health.

## Figures and Tables

**Table 1:** Comparison of key studies on loneliness in COPD.

Study	Country	Sample Size	Population	Measurement Tool	Prevalence of Loneliness
Alqahtani et al., 2024 [7] (Meta-analysis)	Multiple	4644	COPD patients (varied severity)	Mixed tools (UCLA, single-item)	32% (95% CI: 16–48%)
Suen et al., 2023 [16] (HRS)	USA	10,384	General older population (≥50 years), including COPD	3-item UCLA scale	18% (COPD); 22% (oxygen users)
Liu et al. 2025 [17] (UK Biobank)	UK	293,864	General population	Single-item loneliness measure	Loneliness associated with ↑ risk of incident COPD (HR: 1.31; 95% CI: 1.20–1.43)

Abbreviations: CI, confidence interval; HR, hazard ratio; UCLA, University of California, Los Angeles Loneliness Scale. ↑, increased.

## Data Availability

No new data were created or analysed in this study.
